# Editorial: Microbial Utilization and Transformation of Dissolved Organic Matter in Aquatic Environments–From Streams to the Deep Ocean

**DOI:** 10.3389/fmicb.2021.668560

**Published:** 2021-05-13

**Authors:** Johanna Sjöstedt, Jean-Francois Lapierre, Youhei Yamashita, Federico Baltar

**Affiliations:** ^1^Department of Biology, Aquatic Ecology, Lund University, Lund, Sweden; ^2^Department of Biological Sciences, Université de Montréal, Montréal, QC, Canada; ^3^Faculty of Environmental Earth Science, Hokkaido University, Sapporo, Japan; ^4^Department of Functional and Evolutionary Ecology, University of Vienna, Vienna, Austria

**Keywords:** dissolved organic matter, carbon cycling, bacterial communities, ecosystem processes, aquatic environments

Dissolved organic matter (DOM) is the major pool of organic carbon in marine and freshwaters, and the link between detrital organic matter and microorganisms represents one of the largest fluxes in aquatic carbon cycling (Cole, 1999). In order to comprehend aquatic carbon cycling, it is central to understand which factors regulate microbial utilization and transformation of DOM. Evidence suggests that microbial utilization of DOM is influenced by the bacterial community structure but also by the source and composition of DOM (Berggren et al., 2010; Koehler et al., 2012; D'Andrilli et al., 2019). Yet, despite a wide interest in assessing the regulating factors of DOM bioavailability, it is still to a large extent unknown what mechanisms control the ability of microorganisms to utilize DOM and how this changes in different aquatic environments.

Over the last years, methods have been developed for characterizing DOM based on both optical properties (absorbance and fluorescence) (Fellman et al., 2010; Murphy et al., 2013) and high-resolution mass spectrometry (Koch et al., 2007). In addition, advances in next generation sequencing technologies make it possible to reveal both compositional and functional information about microbial communities in an unprecedented manner (Caporaso et al., 2012). Finally, the growing body of literature on the various facets of microbial communities' functioning and DOM cycling has led to a critical mass of published data available for cross-system syntheses. Together, these developments open up possibilities to link microbial communities with the transformation of specific carbon compounds. Although several recent studies have already shown that specific taxa are associated with particular groups of carbon compounds (Covert and Moran, 2001; Foreman and Covert, 2003; Amaral et al., 2016; Logue et al., 2016; Balmonte et al., 2019; Broman et al., 2019), few studies have included functional measurements showing the link to ecosystem process rates. The 15 studies included in this e-book published in *Frontiers in Microbiology* tackle this knowledge gap. They can be grouped in to three main focuses, which we have defined as (1) “Variability of DOM composition and transformations in time and space,” (2) “Transformation of DOM in relation to composition of the DOM pool and/or microbial community,” and (3) “Factors controlling DOM processing and its link to ecosystem processes.”

The first two studies in the category “Variability of DOM composition and transformations in time and space” characterize humic-like DOM based on optical properties. Omori et al. focused on fluorescent DOM (FDOM) in surface water whereas Shigemitsu et al. determined full depth vertical profiles of fluorescent organic matter (FOM) along two meridional transects in the Indian Ocean. Both studies find a linear relationship between FDOM/FOM and oxygen concentration, indicating that FDOM/FOM is produced as the by-products of microbial respiration. In addition, Omori et al. demonstrated diurnal variations in the fluorescence intensities of FDOM due to bacterial production and photobleaching. Furthermore, Shigemitsu et al. concluded that FOM is a possible sink for reduced carbon, estimating the turnover time in the dark Indian Ocean to be 410 ± 19 years.

The two other manuscripts in this category focus on spatial variation in carbon and dissolved organic phosphorus utilization, respectively. Sala et al. explored the utilization of 95 carbon sources by pelagic prokaryotic communities from surface to bathypelagic waters at 111 stations across the tropical and subtropical Atlantic, Indian, and Pacific oceans. The relative use of the individual substrates was remarkably consistent across oceanic regions and layers, and only the equatorial Pacific showed a different metabolic structure. Yamaguchi et al. determined the concentrations of P-esters and compared them to the hydrolysis rates of mono and diesters in the epipelagic North Pacific. P-diester concentrations were generally lower than monoester. Labile diesters and diesterase activities were generally independent of microbial P stress, whereas a tighter link was found between labile monoesters and P stress by large microbes. The studies in this category represent some of the first examples of spatial and temporal patterns of DOM composition and transformations, and therefore an important contribution toward linking microbial communities with DOM utilization.

The manuscripts focusing on “Transformation of DOM in relation to composition of the DOM pool and/or microbial community” are very diverse. The first three studies investigated the microbial response to addition of carbon substrates. Goto et al. found that the production efficiency of bacterial DOM from glucose varied (4–20%) among three marine strains (*Alteromonas macleodii, Vibrio splendidus*, and *Phaeobacter gallaeciensis*). In addition, four processes for the production of bacterially-derived recalcitrant humic-like FDOM were indicated. Liu et al. linked DOM compounds to specific prokaryotic lineages using DNA-stable isotope probing (SIP) experiments with ^13^C-labeled substrates of varying lability. They found that copiotrophs were dominating ^13^C incorporation in the amino acid treatment, and oligotrophs in the more refractory SPE-DOM treatments. Similarly, Pontiller et al. found, in DOM regrowth experiments, that compound class (e.g., carbohydrates, proteins, or nucleic acids) and condensation state were related to bacterial community composition and functional responses. These findings expand results from previous studies by highlighting the specific genes presumably responsible for the processing of specific pools of DOM.

The other manuscripts within this category focus on the link between bacterial community and quality and composition of DOM. Stephens et al. studied dynamics in OM bioavailability at Ocean Station Papa in the North Pacific and its relation to prokaryotic growth efficiency and taxonomy. They found that removal rates of C were higher under more labile DOM composition conditions, and that the prokaryotic taxa utilizing and modifying the DOM composition differed depending on the DOM bioavailability. Varela et al. showed a dynamic interplay between the size of DOM and microbial community structure. Rare prokaryotes from North Atlantic mesopelagic water (e.g., *Alteromonadales*) preferentially utilized high molecular weight-DOM components, while members of the *Rhodobacterales* and *Flavobacteriales* preferentially utilized components of low molecular weight-DOM. Manna et al. provided resident microbial communities organic matter generated from native microplankton to mimic the particle export that may derive from phytoplankton blooms. They found that several rare members became dominant in response to the addition of phytodetritus, and that the characteristic of the organic matter sources caused a specific response in prokaryotic community composition and organic matter utilization. In another incubation experiment, Tinta et al. simulated a scenario of the decay of a bloom of the cosmopolitan *Aurelia aurita s.l*… Sinking jellyfish detrital organic matter (jelly-OM) becomes a significant source of OM for marine microorganisms after jellyfish blooms, and it was estimated that half of the jelly-OM pool is degraded and incorporated into biomass by opportunistic bacteria (*Pseudoalteromonas, Alteromonas*, and V*ibrio*). Hofmann et al. performed long-term (171d) experiments using sediment flow-through microcosms where microbial communities received DOM of different concentrations and compositions. They found that molecularly more diverse DOM caused a higher microbial diversity whereas higher DOM availability resulted in lower carbon use efficiency of aquifer microbial community. In contrast, Kadjeski et al. did not find an effect of DOM source when investigating temporal changes in biodegradability/productivity of DOM in agricultural and forested streams in southern Ontario, Canada. Based on incubation experiments they found that biodegradability and productivity of DOM were the same in both streams and synchronous throughout the sampling period, even though a more allochthonous-like DOM signature and a more autochthonous-like DOM signature dominated in the forested and agricultural streams, respectively. In summary the manuscripts from this category show that the utilization and bioavailability of DOM should be seen as an interaction between the chemical composition of DOM and the metabolic capacity of the microbial community.

In the last category “Factors controlling DOM processing and its link to ecosystem processes,” Allesson et al. combined gradient lake surveys with laboratory experiments to show that the DOC concentration regulates the overall respiratory output of CO_2_ while additions of P and increased temperature change the dynamics between respiration and growth. While total CO_2_ production seemed to be unaffected by P additions, respiration rates and growth yields suggested increased bacterial growth and decreased cell-specific respiration under non-limited P conditions.

Finally, the perspective article by Baltar et al. provides an updated description of refractory DOC (rDOC) addressing the problem of various definitions and approaches currently used to characterize rDOC. Based on previous evidence, they conclude that the persistence of rDOC is mostly a function of molecular properties, molecular concentrations, and ecosystem properties. By deepening the connection between DOM and microbes, from the individual compounds to an ecosystem scale, this perspective provides a stimulating conclusion to the Research Topic.

We hope that this collection of articles will stimulate further discussions. Although many challenges and questions remain to be addressed, the articles published in this e-book represent a significant advance in our understanding of microbial utilization and transformation of DOM in aquatic environments, especially considering the link between DOM and microbial community composition. However, there is still limited data and studies on ecosystem process rates. We therefore suggest that future research should include functional measurements based on either omics or measurements of process rates (e.g., bacterial production, enzyme activity, or bacterial respiration), ideally combined with DOM characterization.

## Author Contributions

All authors contributed to the writing of the editorial.

## Conflict of Interest

The authors declare that the research was conducted in the absence of any commercial or financial relationships that could be construed as a potential conflict of interest.
